# Temporal dynamics of co‐inoculation of *Leptosphaeria maculans* and *Leptosphaeria biglobosa* determine interspecific competition and disease development on oilseed rape

**DOI:** 10.1002/ps.70643

**Published:** 2026-02-08

**Authors:** Evren Bingol, Chinthani S. Karandeni Dewage, Aiming Qi, Faye Ritchie, Bruce DL Fitt, Yong‐Ju Huang

**Affiliations:** ^1^ School of Health, Medicine and Life Sciences University of Hertfordshire Hatfield UK; ^2^ Department of Medicine, Public Health and Life Sciences Disease and Pest Management, ADAS Boxworth Cambridge UK

**Keywords:** disease severity, interspecific competition, *Leptosphaeria* biglobosa, *Leptosphaeria maculans*, oilseed rape, phoma stem canker

## Abstract

**BACKGROUND:**

Phoma stem canker is one of the most economically damaging diseases of oilseed rape, caused by co‐existing fungal pathogens *Leptosphaeria maculans* (*Plenodomus lingam*) and *Leptosphaeria biglobosa* (*Plenodomus biglobosus*). Our previous work showed that timing of their co‐inoculation affects their interspecific interactions *in vitro*, and that *L. biglobosa* can inhibit the growth of *L. maculans* if it is inoculated before or up to 1 day after *L. maculans*. However, the effects of timing of co‐inoculation on their interspecific interactions within host plants are not fully understood.

**RESULTS:**

The interactions between *L. maculans* and *L. biglobosa* were investigated *in planta* by inoculation with *L. maculans* first, followed by *L. biglobosa* sequentially 1, 3 or 5 days later or vice versa, or *L. maculans* and *L. biglobosa* co‐inoculated simultaneously; controls were inoculation with *L. maculans* only, *L. biglobosa* only or sterilised distilled water. The results showed that *L. biglobosa* can inhibit the growth of *L. maculans* locally if it is inoculated before or up to 1 day after *L. maculans in planta*.

**CONCLUSION:**

The results of this study provide evidence that the time *L. maculans* and *L. biglobosa* meet and interact on their host significantly affects the interspecific competition and subsequent disease severity. *Leptosphaeria biglobosa* can deploy multiple interspecific competition strategies against *L. maculans* to have local and systemic effects on the host to outcompete *L. maculans*. There is a need to investigate and model the factors affecting the timing of ascospore release and subsequent interactions to develop better control strategies. © 2026 The Author(s). *Pest Management Science* published by John Wiley & Sons Ltd on behalf of Society of Chemical Industry.

## Graphical abstract

This study provides evidence that the timing of co‐inoculation of *Leptosphaeria maculans* and *Leptosphaeria biglobosa* determine the outcome of interspecific competition and disease development on their host oilseed rape.

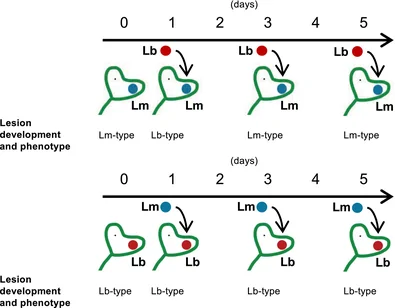

## 1 INTRODUCTION

Crops in nature are faced with a variety of pests and pathogens, often plant pathogenic fungi that pose a threat to food security.^1^, ^2^ Oilseed rape (*Brassica napus*) has over 15 pathogens globally, each affecting different parts of the plant during its life cycle in a growing season.^3^ Phoma stem canker (blackleg) is one of the most serious threats to oilseed rape (canola) crops worldwide.^4^ Considering just the United Kingdom (UK), this disease causes annual yield losses > £80 million, in addition to disease control efforts by fungicide applications costing approximately another £20 million.^5^, ^6^, ^7^ The causal agents of phoma stem canker are two closely related fungal phytopathogens *Leptosphaeria maculans* (*Plenodomus lingam*) and *Leptosphaeria biglobosa* (*Plenodomus biglobosus*).^8^, ^9^, ^10^ Throughout Europe, phoma stem canker epidemics start in the autumn, with the release of ascospores from pseudothecia developed on crop debris from the previous growing season.^11^, ^12^ These ascospores land and germinate on leaf surfaces, and hyphae penetrate through stomata and cause leaf spot lesions.^13^, ^14^ This is followed by hyphal growth of the pathogens along the leaf petiole, down to the stem, resulting in crown cankers the following summer before harvest.^15^, ^16^ *Leptosphaeria maculans* is associated with large, grey leaf lesions with pycnidia and stem basal cankers, which are considered more damaging; whereas *L. biglobosa* is associated with small, black leaf lesions with very few or no pycnidia and upper stem lesions, which are considered less damaging.^9^, ^17^ However, there are recent reports of *L. biglobosa* being frequently detected in stem basal cankers.^18^, ^19^, ^20^ Furthermore, only *L. biglobosa* is found in China and it can cause significant yield losses.^6^, ^21^, ^22^ *Leptosphaeria maculans* and *L. biglobosa* are found to co‐infect oilseed rape crops in Europe, Canada and Australia.^23^, ^24^

Co‐infection of a single host by multiple pathogen species can result in interspecific interactions, which are classified in three groups: competition, co‐operation or co‐existence.^1^, ^25^ Interspecific competition can result in niche specialisation to facilitate co‐existence, if allowed by the biology of the pathogens. Co‐existence of *L. maculans* and *L. biglobosa* on oilseed rape is facilitated by the temporo‐spatial gap between the release of their ascospores, leading to small differences in the ecological niches they occupy.^9^, ^23^ This is due to the differences in optimal temperatures required for pseudothecial maturation between these pathogens. Pseudothecia of *L. maculans* mature faster than those of *L. biglobosa* at lower temperatures (5–10 °C), leading to release of *L. maculans* ascospores earlier in the growing season compared to those of *L. biglobosa*.^9^, ^23^, ^26^ However, recent studies reported that ascospores of both *L. maculans* and *L. biglobosa* are being released at similar times in the UK, and that the frequency of *L. biglobosa* in stem basal cankers is increasing, especially in cultivars with functional resistance genes against *L. maculans*.^20^ These factors may lead to interspecific competition arising between *L. maculans* and *L. biglobosa*.

When interspecific competition takes place, the pathogens can deploy three different strategies.^1^ Firstly, in resource‐mediated (or exploitative) competition strategy, when a resource becomes limited, the species that can utilise the resource most efficiently for growth and development would outcompete the others to survive. This can sometimes lead to competitive exclusion of other species.^27^, ^28^ Secondly, in host‐mediated (or apparent) competition strategy, the infection of the host by one of the competitors activates the plant defence systems against other competing pathogens, leading to a reduction in their chances of successful infection.^29^ Finally, in interference competition strategy, one of the competitors interferes with another's access to a limited resource. This is often achieved by the production of toxins.^1^ Some of these interspecific competition strategies have been observed between *L. maculans* and *L. biglobosa*.

Previous studies showed that pretreatment of oilseed rape leaves with *L. biglobosa* ascospores induced systemic acquired resistance (SAR) by activating the oilseed rape defence responses and reduced the severity of subsequent disease caused by *L. maculans*.^30^, ^31^ Interestingly, this effect of *L. biglobosa* was lost if it was inoculated 64 h or later after *L. maculans*,^30^ which suggests that the timing of their interaction impacts their competition and can subsequently affect disease severity. Further research has investigated the expression of genes in relation to activation of plant immune signalling pathways.^31^, ^32^ These pathways are activated by phytohormones including salicylic acid (SA) and jasmonic acid (JA)/ethylene (ET), which are related to defence responses against hemibiotrophic and necrotrophic plant pathogens, respectively.^33^, ^34^ Moreover, SAR is usually associated with the *PR‐1* gene, which is also associated with the SA pathway.^35^, ^36^ In addition, some *WRKY* transcriptions factors were reported to be important regulators of these pathways, with *WRKY33* being especially important for defence against necrotrophic pathogens.^37^, ^38^

Previous work reported that the phytotoxin sirodesmin PL produced by *L. maculans* had an inhibitory effect on the growth of an *L. biglobosa* colony towards *L. maculans in vitro*, resulting in a zone of inhibition. This was confirmed when a mutant sirodesmin PL non‐producing isolate of *L. maculans* did not have an inhibitory effect on *L. biglobosa*.^39^ Sirodesmin PL is a non‐host specific epipolythiodioxopiperazine (ETP) phytotoxin, which has antibiotic and antifungal properties.^39^, ^40^, ^41^ Conversely, further work showed that *L. biglobosa* can inhibit the production of sirodesmin PL by *L. maculans* both *in vitro* and *in planta*, if they were co‐inoculated simultaneously.^42^ However, this inhibition did not take place if *L. biglobosa* was co‐inoculated 7 days after *L. maculans*. This also suggests that the timing when *L. maculans* and *L. biglobosa* meet and interact affects the outcome of their interspecific competition.

Recent work investigated the effects of co‐inoculation timing on interspecific interactions between *L. maculans* and *L. biglobosa in vitro*.^43^ It was found that *L. biglobosa* was not only able to inhibit the production of sirodesmin PL but it was also able to inhibit the growth of *L. maculans*. However, this was found to be dependent on the timeline of their co‐inoculation; with *L. biglobosa* able to partially inhibit the growth and sirodesmin PL production by *L. maculans* if co‐inoculated 3 days after but losing all its inhibitory effects on *L. maculans* if co‐inoculated 5 days after or later.^43^ This was attributed to the requirement of 3–5 days before *L. maculans* can produce sirodesmin PL *in vitro*.^41^, ^42^ We hypothesised that timing of co‐inoculation would affect the interspecific competition between *L. maculans* and *L. biglobosa* similarly *in planta*. The aims of this research were to investigate the effects of sequential co‐inoculation on interspecific interactions between *L. maculans* and *L. biglobosa* in terms of lesion development, pycnidial production, relative growth of the pathogens and host defence responses, and the potential impact of the interspecific interactions on disease management.

## 2 MATERIALS AND METHODS

### 2.1 Preparation of inoculum and plants

The isolates used in this study are *L. maculans* ‘brassicae’ isolate ME24, obtained from phoma stem canker on oilseed rape cultivar Apex in 2002 (UK)^15^ and *L. biglobosa* ‘brassicae’ isolate WH17 Why‐1, obtained from phoma leaf spot of oilseed rape cultivar Whisky in 2017 (UK).^42^ The *L. maculans* isolate ME24 carries three avirulence (*Avr*) genes (*AvrLm1, AvrLm6* and *AvrLm7*). *Leptosphaeria maculans* and *L. biglobosa* conidial suspensions for inoculation were obtained from 10‐day‐old cultures on 20% V8 agar.^15^ The concentrations of conidial suspensions were measured and adjusted to 1 × 10^7^ conidia mL^−1^. Oilseed rape cultivar Charger (with no known resistance genes) was selected for inoculation. Plants were grown by sowing pre‐germinated seeds into 40‐well plug trays containing a 1:1 ratio of John Innes Number 3 soil (Westland Horticulture Ltd, Standroyd Mill, Cotton Tree Lane, Colne, Lanc, UK) and MiracleGro multipurpose compost (MiracleGro Company, B&M Home Store, Retail Park, Unit 2 Oldings Cor, Hertfordshire, AL9 5JP). The plug trays were placed in a controlled environment cabinet with 20 °C/18 °C and 12 h:12 h day/night. Plants were watered regularly, and emerging true leaves were removed to increase the lifespan of cotyledons.

### 2.2 Plant inoculation

To investigate the lesion phenotype and development, pycnidial production, and relative amounts of *L. maculans* and *L. biglobosa* DNA in lesions, two replicated experiments with ten treatments were done. In each experiment, the ten treatments were: sterilised distilled water (SDW) for control, *L. maculans* only (Lm only), *L. biglobosa* only (Lb only), *L. maculans* and *L. biglobosa* simultaneous co‐inoculation (Lm&Lb), initial inoculation with *L. maculans* followed by *L. biglobosa* 1, 3 or 5 days later (Lm + Lb‐1, Lm + Lb‐3, Lm + Lb‐5), and initial inoculation with *L. biglobosa* followed by *L. maculans* 1, 3 or 5 days later (Lb + Lm‐1, Lb + Lm‐3, Lb + Lm‐5). There were 15 plants per treatment and the experiments were done using a randomised block design with three replicates. To investigate the host defence responses, two replicated experiments with eight treatments were done. The eight treatments were: SDW for control, *L. maculans* only (Lm only), *L. biglobosa* only (Lb only), *L. maculans* and *L. biglobosa* simultaneous co‐inoculation (Lm&Lb), initial inoculation with *L. maculans* followed by *L. biglobosa* 3 or 5 days later (Lm + Lb‐3, Lm + Lb‐5), and initial inoculation with *L. biglobosa* followed by *L. maculans* 3 or 5 days later (Lb + Lm‐3, Lb + Lm‐5). For the treatments ‘SDW’, ‘Lm only’, ‘Lb only’ and ‘Lm&Lb’, there were ten plants per treatment with four replicate blocks. For the treatments ‘Lm + Lb‐3’, ‘Lb + Lm‐3’, ‘Lm + Lb‐5’ and ‘Lb + Lm‐5’, there were ten plants per treatment with two replicate blocks.

Cotyledons of 14‐day‐old plants were point‐inoculated, using the puncture wound method.^44^ Using a heat‐sterilised microscope needle, cotyledons were wounded (one wound per lobe, two wounds per cotyledon, four wounds per plant). For inoculation, 11 μL of conidial suspension was pipetted onto each wound according to the treatments. Inoculum for the ‘Lm&Lb’ treatment was obtained by mixing conidial suspensions of each *Leptosphaeria* species in a 1:1 ratio. Following inoculation, the plants were covered with misted propagator lids and kept in darkness for the first 24 h. The propagator lids were kept for another 48 h (total 72 h) to maintain high humidity. Inoculated plants were maintained in a controlled environment cabinet with 18 °C/16 °C and 12 h:12 h day/night.

### 2.3 Assessment of lesion development and pycnidial production

For the experiments with ten treatments, plants were assessed regularly after inoculation. Lesion development was assessed by measuring the lesion area (length by width) at 7, 10, 14 and 16 days post‐inoculation (dpi). Lesions at inoculation sites per treatment were measured at each time point in each experiment. After the final assessment at 16 dpi, two cotyledons per treatment were randomly sampled and placed on 2% water agar with the lid misted with sterilised distilled water. They were incubated at 20 °C in darkness for 48 h to promote pycnidial production. Using a Nikon YS100 light microscope at 100× magnification, areas near the inoculation sites of the incubated cotyledons were viewed and photographed using a smartphone camera. The number of mature pycnidia (with circular pink cirri containing conidia) in a view area of 0.1 mm^2^ were counted to calculate the density of pycnidia production.

### 2.4 DNA extraction from cotyledons

After the final assessment of lesion area, six cotyledons per treatment were randomly sampled and placed in sterile 2 mL screw‐cap tubes and stored at −20 °C before freeze‐drying for 48 h. DNA extractions from freeze‐dried cotyledons were done using the sorbitol pre‐wash protocol,^45^ with sufficient modifications.

Three sterile stainless‐steel beads (3 mm diameter) were added into each tube containing a cotyledon sample and processed using a FastPrep machine at 4.0 m s^−1^ for 20 s. This was followed by adding 1.5 mL of sorbitol pre‐wash buffer, vortexing for 5 s, and centrifugation at 5000 × *g* for 5 min at 20 °C. Following centrifugation, the supernatant containing polysaccharides, polyphenols and pigments was aspirated using a pipette. Then, 700 μL of lysis buffer (pre‐warmed to 65 °C) was added to each tube and vortexed for 5 s. The tubes were then placed in a 65 °C water bath for 40 min and mixed by inverting five times every 10 min. After cooling the samples at 20 °C for 5 min, 700 μL of chloroform/isoamyl alcohol (24:1) was added into each tube and vortexed for 10 s, followed by centrifugation at 5000 × *g* for 10 min at 20 °C. The upper aqueous phase (700 μL) was transferred into new sterile 1.5 mL tubes, and 70 μL of sodium acetate (3 m, pH 5.2, at 4 °C) and 460 μL of isopropanol (at −20 °C) were added. These tubes were mixed by inverting ten times and incubated at −20 °C for 1 h. The tubes were then centrifuged at 13 000 × *g* for 10 min at 20 °C and the supernatant was discarded. This was followed by adding 700 μL of ethanol (70%, at −20 °C) and centrifugation at 13 000 × *g* for 10 min at 20 °C. The supernatant was discarded, and any remnants were aspirated using a pipette. After drying in a fume hood for 30 min, the DNA pellets were re‐suspended in 30 μL of nuclease‐free water, incubated at 4 °C for 1 h and then stored at −20 °C.

DNA concentration of each sample was measured using Qubit DNA Broad Range Assay Kit (Invitrogen, Carlsbad, CA, USA) and diluted to 20 ng μL^−1^ for quantitative polymerase chain reaction (qPCR) analyses.

### 2.5 Quantification of pathogen DNA in lesions

To assess the relative growth of *L. maculans* and *L. biglobosa* in different treatments, the amounts of pathogen DNA were quantified using SYBR Green (Agilent, Santa Clara, CA, USA), with species‐specific internal transcribed spacer (ITS) primers LmacF (5′‐CTTGCCCACCAATTGGATCCCCTA‐3′)/LmacR (5′‐GCAAAATGTGCTGCGCTCCAGG‐3′) (for *L. maculans*) and LbigF (5′‐ATCAGGGGATTGGTGTCAGCAGTTGA‐3′)/LmacR (5′‐GCAAAATGTGCTGCGCTCCAGG‐3′) (for *L. biglobosa*).^46^ The qPCR reactions were prepared at a total volume of 20 μL, containing 10 μL of SYBR Green (containing ROX as a reference dye), 6.3 μL of nuclease‐free water, 0.6 μL of forward primer (10 μm), 0.6 μL of reverse primer (10 μm), and 2.5 μL of DNA sample in duplicate in 96‐well plates. To produce standard curves, ten‐fold dilution series (i.e., six concentration points from 10^4^ pg to 10^−1^ pg) of *L. maculans* and *L. biglobosa* DNA obtained from pure cultures were used.^47^ The amounts of *L. maculans* and *L. biglobosa* DNA in samples were calculated using the standard curves with the CFX Maestro software (Bio‐Rad, Hercules, CA, USA).

The qPCR reactions were done using a CFX Opus 96 Real‐Time PCR System (Bio‐Rad). Thermal cycling profiles for the reactions were one cycle of 95 °C for 2 min, 40 cycles of 95 °C for 15 s, 60 °C (for *L. maculans*) or 55 °C (for *L. biglobosa*) for 30 s, 72 °C for 45 s, and a reading step of 83 °C for 15 s. Melting curve analysis was done by increasing the temperature from 60 °C (for *L. maculans*) or 55 °C (for *L. biglobosa*) to 95 °C at 0.5 °C increments at 3 s per step.

### 2.6 Gene expression analyses

At 2, 4 and 6 dpi, four cotyledons (each cotyledon from an individual plant) were randomly selected, removed, placed in 2 mL screw‐cap tubes and immediately placed in liquid nitrogen before storing at −80 °C. RNA extractions were done using the RNeasy Plant Mini Kit (Qiagen, Venlo, The Netherlands), according to the manufacturer's guidelines, with minor modifications. One Zirconia bead (cooled in liquid nitrogen) was added into each tube. The samples were homogenised by processing in the TissueLyser system at 30 Hz for 20 s, 450 μL of Buffer RLT was added, and the samples were processed again using the same settings. Lysates were transferred to QIAshredder columns and centrifuged at 15 700 × *g* for 2 min at 20 °C. The flow‐throughs were taken into sterile 1.5 mL tubes and mixed with 0.5 volume of 100% ethanol using a pipette. The samples were then transferred into RNeasy spin columns and centrifuged at 10 000 × *g* for 15 s at 20 °C. The flow‐throughs were discarded, and the membranes of the columns were washed with 700 μL of Buffer RW1 and centrifuging using the same settings. This was followed by washing the membranes with 500 μL of Buffer RPE twice, with a longer centrifugation time of 2 min for the second wash. After discarding the flow‐throughs, the columns were placed in sterile 1.5 mL tubes and the RNA was eluted by adding 50 μL of nuclease‐free water directly into the membrane and centrifuging at 10 000 × *g* for 1 min at 20 °C.

RNA concentration of each sample was measured using a Qubit RNA Broad Range Assay Kit (Invitrogen). DNase treatment was done using the RQ1 RNase‐Free DNase Kit (Promega, Madison, WI, USA), according to the manufacturer's guidelines, where 1 μL each of RQ1 reaction buffer and RQ1 RNase‐free DNase were combined with 1 μg of RNA (diluted in DEPC‐treated water up to a maximum volume of 8 μL per sample). After incubating at 37 °C for 30 min using a thermocycler, 1 μL of RQ1 stop solution was added into each sample, followed by incubation at 65 °C for 10 min. Complementary DNA (cDNA) synthesis was done using the UltraScript 2.0 Reverse Transcriptase Kit (PCR Biosystems, Wayne, PA, USA), according to the manufacturer's guidelines, where 4 μL of cDNA synthesis mix and 1 μL of UltraScript 2.0 were added into each sample tube following DNase treatment. This was followed by incubating the samples at 55 °C for 30 min, then 95 °C for 10 min.

To assess the relative expression of pathogenesis‐related protein (*PR‐1*) and WRKY transcription factor 33 (*WRKY33*) genes in cotyledons inoculated with different treatments, the transcripts were quantified using SYBR Green (Agilent), with the primers PR‐1‐F (5′‐GGCTAACTATAACCACGATTC‐3′)/PR‐1‐R (5′‐GTTCCACCATTGTTACACC‐3′) and WRKY33‐F (5′‐TGTCGGACAGCTTGGGAAAG‐3′)/WRKY33‐R (5′AGAGGACGGTTACAACTGGAGAAA‐3′). Actin was used as a reference gene with the primers ACTIN‐F (5′‐CTGGAATTGCTGACCGTATGAG‐3′)/ACTIN‐R (5′‐GTTGGAAAGTGCTGAGGGATG‐3′).^32^ The qPCR reactions were prepared at a total volume of 10 μL, containing 5 μL of SYBR Green, 2.2 μL of nuclease‐free water, 0.4 μL of forward primer (10 μm), 0.4 μL of reverse primer (10 μm), and 2 μL of 1:10 diluted cDNA sample in triplicate wells in 96‐well plates.

The qPCR reactions were done using a CFX Opus 96 Real‐Time PCR System (Bio‐Rad). Thermal cycling profiles were one cycle of 95 °C for 2 min, 40 cycles of 95 °C for 5 s, 60 °C for 10 s and a reading step of 72 °C for 30 s. This was followed by a melting curve analysis by increasing the temperature from 60 to 95 °C in 0.5 °C increments at 3 s per step. The relative expression of genes was calculated using the 2^−ΔΔCt^ method.^48^

### 2.7 Statistical analysis

All statistical analyses were done using GraphPad Prism (version 10 for macOS) (GraphPad Software, Boston, MA, USA). To compare the differences in lesion areas between different treatments, the data were transformed using a natural logarithm to homogenise the variances. Then a simple linear equation of *y* = *a* + *bx* was fitted to describe the relationship between the average lesion area and time post‐inoculation for each treatment. In this equation, *y* is the average lesion area (in mm^2^), *x* is in days post‐inoculation, *a* is the parameter intercept and *b* is the parameter slope quantifying the rate of lesion development. The rates of lesion development in different treatments were then analysed by comparing the slopes of the equations using an analysis of variance (ANOVA). Tukey *post hoc* tests were used to separate the means of the treatments at 5% significant probability (*P* < 0.05).

To compare the differences between the ten different treatments in lesion areas at 16 dpi, pycnidial production and relative pathogen DNA in lesions, an ANOVA was done. To compare the differences in the expression of *PR‐1* and *WRKY33* between the four different treatments, the data were transformed using a natural logarithm to homogenise the variances, followed by ANOVA. Since two replicated experiments were done, experiment was assigned as a factor when analysing the data to assess whether the experiment had a significant effect. The *F*‐values that were significant at *P* < 0.05 indicated significant effects of the factors. Tukey *post hoc* tests were used to separate the means of the treatments at 5% significant probability.

## 3 RESULTS

### 3.1 Comparison of leaf lesion development

There were significant differences in lesion phenotype between different treatments. Lesion phenotype for ‘Lm + Lb‐3’ and ‘Lm + Lb‐5’ were larger, grey and less defined, similar to ‘Lm only’, whereas lesion phenotype for ‘Lm&Lb’, ‘Lm + Lb‐1’, ‘Lb + Lm‐1’, ‘Lb + Lm‐3’ and ‘Lb + Lm‐5’ were smaller, darker and more defined, similar to ‘Lb only’ (Fig. 1).

**Figure 1:**
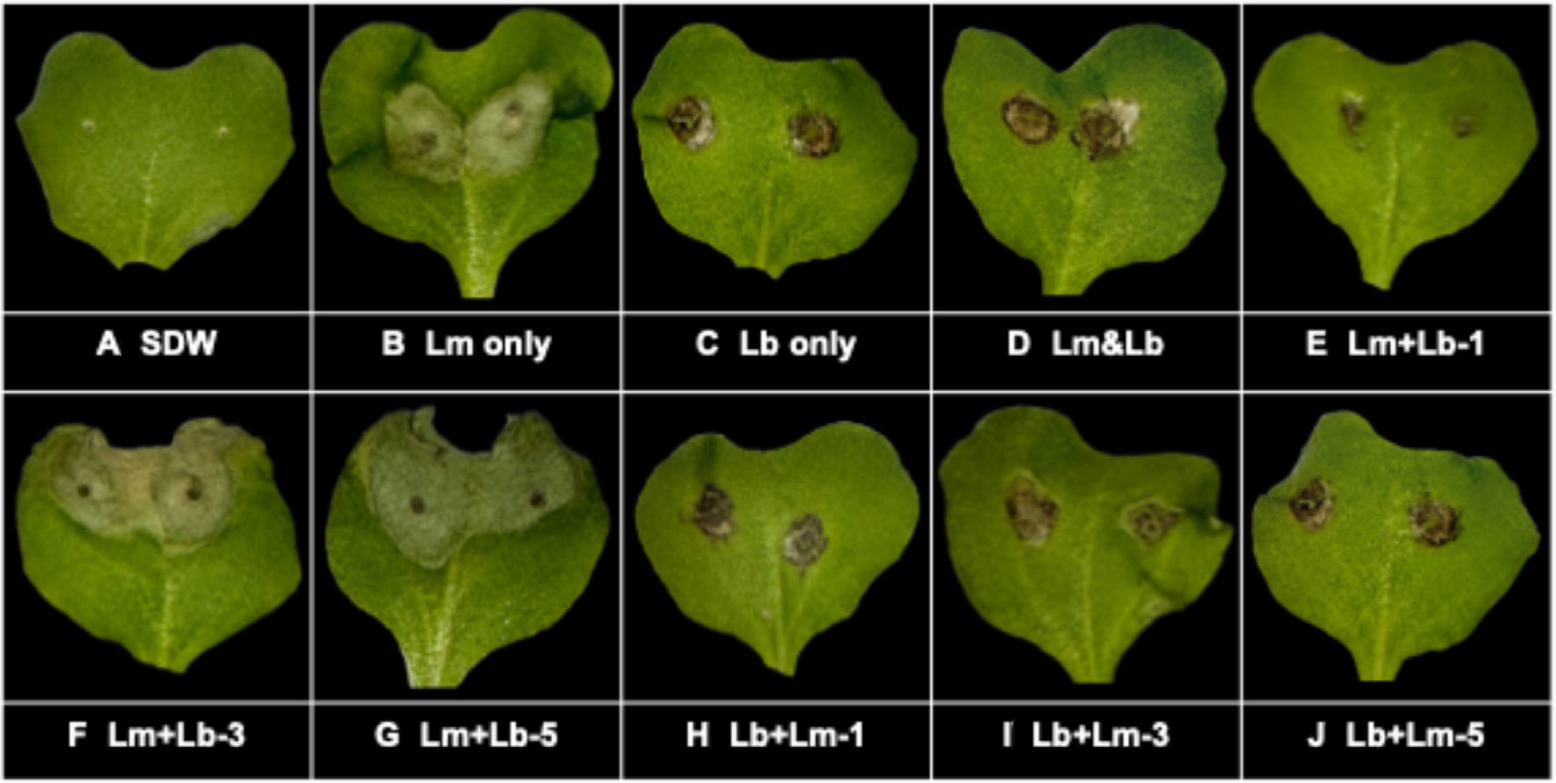
Lesion phenotypes for different treatments. Cotyledons of oilseed rape (*Brassica napus*) cultivar Charger were inoculated with (A) sterilised distilled water (SDW) for control, (B) *Leptosphaeria maculans* only (Lm only), (C) *Leptosphaeria biglobosa* only (Lb only), (D) *L. maculans* and *L. biglobosa* co‐inoculated simultaneously (Lm&Lb), (E, F, G) initial inoculation with *L. maculans* followed by co‐inoculation with *L. biglobosa* at 1, 3 or 5 days later (Lm + Lb‐1, Lm + Lb‐3, Lm + Lb‐5) or (H, I, J) initial inoculation with *L. biglobosa* followed by co‐inoculation with *L. maculans* at 1, 3 or 5 days later (Lb + Lm‐1, Lb + Lm‐3, Lb + Lm‐5). Cotyledons were photographed at 14 days post‐inoculation (dpi).

At 16 dpi, there were significant differences between different treatments in the lesion area (*F*(9,44) = 57.38, *P* < 0.0001). The treatments ‘Lm only’ (67.1 mm^2^), ‘Lm + Lb‐3’ (63.6 mm^2^) and ‘Lm + Lb‐5’ (73.4 mm^2^) had significantly larger lesions than all other treatments but were not significantly different from each other (Fig. 2). The treatment ‘Lm + Lb‐1’ (20.6 mm^2^) had significantly smaller lesions than all other treatments. The treatments ‘Lb only’ (45.0 mm^2^), ‘Lm&Lb’ (44.5 mm^2^), ‘Lb + Lm‐1’ (45.8 mm^2^), ‘Lb + Lm‐3’ (51.0 mm^2^) and ‘Lb + Lm‐5’ (50.1 mm^2^) were not significantly different from each other. There were no significant interactions between experiment and treatment (*F*(9,44) = 0.04, *P* = 0.999).

**Figure 2:**
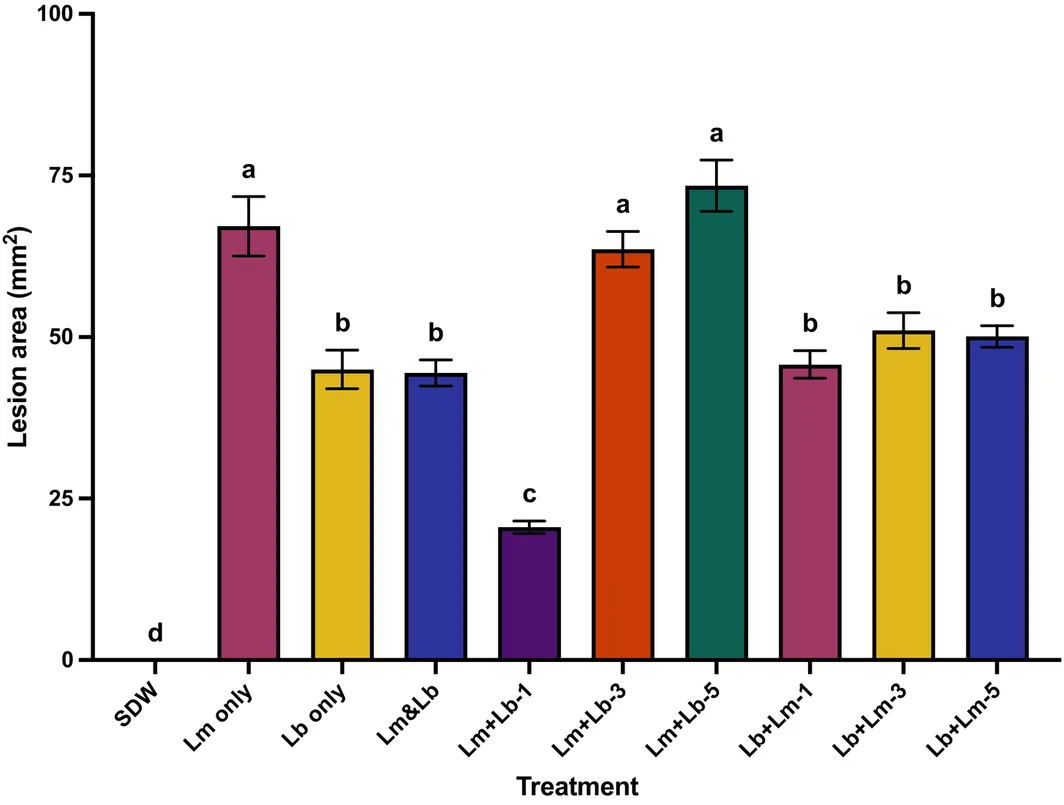
Average lesion area (mm^2^) from two experiments for different treatments. Cotyledons of oilseed rape (*Brassica napus*) were inoculated with sterilised distilled water (SDW, control), *Leptosphaeria maculans* only (Lm only), *Leptosphaeria biglobosa* only (Lb only), *L. maculans* and *L. biglobosa* co‐inoculated simultaneously (Lm&Lb), initial inoculation with *L. maculans* followed by co‐inoculation with *L. biglobosa* at 1, 3 or 5 days later (Lm + Lb‐1, Lm + Lb‐3, Lm + Lb‐5) or initial inoculation with *L. biglobosa* followed by co‐inoculation with *L. maculans* at 1, 3 or 5 days later (Lb + Lm‐1, Lb + Lm‐3, Lb + Lm‐5). Final lesion area was measured at 16 days post‐inoculation (dpi). Tukey's honestly significant difference (HSD) tests were used to compare the mean lesion area between different treatments. Treatments that do not share a letter are considered significantly different (*P* < 0.05). Error bars show standard error of the mean (SEM) (44 df).

There were significant differences in the rates of lesion development (7 dpi to 16 dpi) between different treatments (*F*(9,236) = 47.67, *P* < 0.0001). The lesion development rates were the slopes of the linear equations fitted for each treatment (Table 1). The rates of lesion development for treatments ‘Lm only’ (0.42), ‘Lm + Lb‐3’ (0.40) and ‘Lm + Lb‐5’ (0.43) were significantly greater than those for all other treatments but were not significantly different from each other. The rates of lesion development for treatments ‘Lb only’ (0.24), ‘Lm&Lb’ (0.25), ‘Lm + Lb‐1’ (0.26), ‘Lb + Lm‐1’ (0.27), ‘Lb + Lm‐3’ (0.25) and ‘Lb + Lm‐5’ (0.26) were not significantly different from each other (Table 1).

**Table 1:** The estimated parameter values of coefficient of determination (*R* ^2^), intercept (*a*) and slope (*b*) in the simple linear equation *y* = *a* + *bx* fitted to describe the relationship between the natural logarithm transformed lesion area and days post‐inoculation (dpi) from two experiments for different treatments

Treatment	*R* ^2^ Value	Intercept (*a*)	Slope (*b*)	Slope value comparisons
SDW	0.00	0.00	0.00	c
Lm only	0.91	−2.79	0.42	a
Lb only	0.91	−0.05	0.24	b
Lm&Lb	0.96	−0.09	0.25	b
Lm + Lb‐1	0.91	−1.33	0.26	b
Lm + Lb‐3	0.90	−2.61	0.40	a
Lm + Lb‐5	0.92	−2.88	0.43	a
Lb + Lm‐1	0.97	−0.46	0.27	b
Lb + Lm‐3	0.97	−0.07	0.25	b
Lb + Lm‐5	0.98	−0.15	0.26	b

*Note*: Cotyledons of oilseed rape (*Brassica napus*) were inoculated with sterilised distilled water (SDW, control), *Leptosphaeria maculans* only (Lm only), *Leptosphaeria biglobosa* only (Lb only), *L. maculans* and *L. biglobosa* co‐inoculated simultaneously (Lm&Lb), initial inoculation with *L. maculans* followed by co‐inoculation with *L. biglobosa* at 1, 3 or 5 days later (Lm + Lb‐1, Lm + Lb‐3, Lm + Lb‐5) or initial inoculation with *L. biglobosa* followed by co‐inoculation with *L. maculans* at 1, 3 or 5 days later (Lb + Lm‐1, Lb + Lm‐3, Lb + Lm‐5). Lesion areas were assessed at 7, 10, 14 and 16 dpi. To compare the rate of lesion development, line parallel comparisons were done by using Tukey's honestly significant difference (HSD) tests to compare the mean slope values between different treatments. Treatments that do not share a letter are considered significantly different (*P* < 0.05).

### 3.2 Comparison of pycnidial production on leaf lesions

The production of pycnidia on leaf lesions was assessed by incubating cotyledons with lesions for 48 h in darkness with high humidity. The density of pycnidia was expressed as number of mature pycnidia per 0.1 mm^2^ leaf area. There were significant differences between different treatments in density of mature pycnidia (*F*(9,40) = 550.40, *P* < 0.0001). There were no mature pycnidia observed for the treatments ‘Lb only’, ‘Lm&Lb’, ‘Lm + Lb‐1’, ‘Lb + Lm‐1’, ‘Lb + Lm‐3’ and ‘Lb + Lm‐5’ (Fig. 3). Conversely, mature pycnidia were observed for the treatments ‘Lm only’, ‘Lm + Lb‐3’ and ‘Lm + Lb‐5’ (Fig. 3). There were no significant differences in density of mature pycnidia between treatments ‘Lm only’ (37 pycnidia mm^2^ × 10^−1^), ‘Lm + Lb‐3’ (35 pycnidia mm^2^ × 10^−1^) and ‘Lm + Lb‐5’ (36 pycnidia mm^2^ × 10^−1^). There were no significant interactions between experiment and treatment (*F*(9,40) = 0.47, *P* = 0.885).

**Figure 3:**
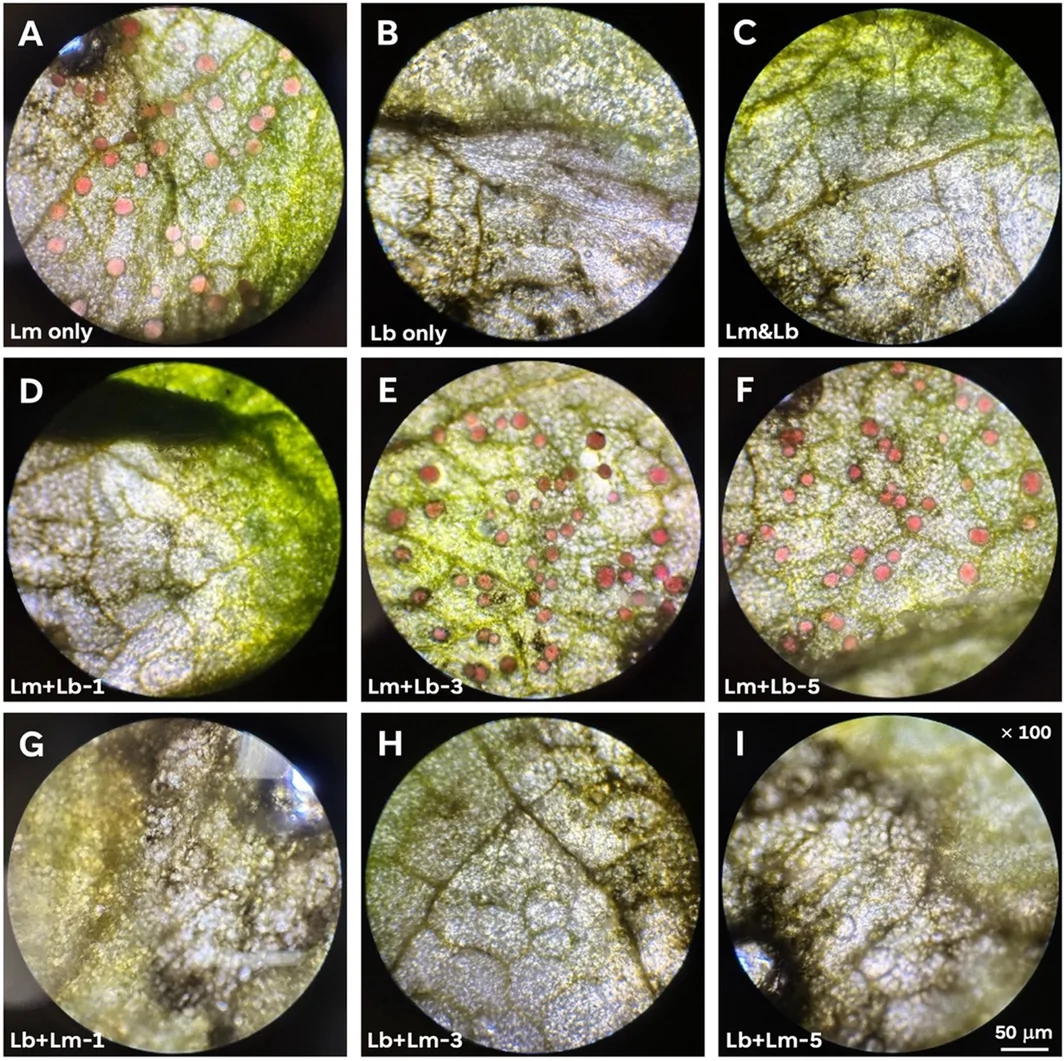
Microscopy images (at 100× magnification) of lesion areas (0.1 mm^2^) near the inoculation site showing production of pycnidia in different treatments. Cotyledons of oilseed rape (*Brassica napus*) cultivar Charger were inoculated with (A) *Leptosphaeria maculans* only (Lm only), (B) *Leptosphaeria biglobosa* only (Lb only), (C) *L. maculans* and *L. biglobosa* co‐inoculated simultaneously (Lm&Lb), (D, E, F) initial inoculation with *L. maculans* followed by co‐inoculation with *L. biglobosa* at 1, 3 or 5 days later (Lm + Lb‐1, Lm + Lb‐3, Lm + Lb‐5) or (G, H, I) initial inoculation with *L. biglobosa* followed by co‐inoculation with *L. maculans* at 1, 3 or 5 days later (Lb + Lm‐1, Lb + Lm‐3, Lb + Lm‐5). Cotyledons were removed at 16 days post‐inoculation (dpi) and incubated at 20 °C in darkness for 48 h before microscopic assessment.

### 3.3 Comparison of pathogen DNA in leaf lesions

The relative growth of *L. maculans* and *L. biglobosa* in cotyledons with leaf lesions was assessed by measuring the amounts of pathogen DNA using qPCR. The amounts of *L. maculans* or *L. biglobosa* DNA in a total of 50 ng of DNA extracted from inoculated cotyledons were expressed as percentages. There were significant differences between different treatments in percentage of *L. maculans* DNA in lesions (*F*(8,70) = 18 648, *P* < 0.0001). The treatments ‘Lb only’, ‘Lm&Lb’, ‘Lm + Lb‐1’, ‘Lb + Lm‐1’, ‘Lb + Lm‐3’ and ‘Lb + Lm‐5’ had significantly less *L. maculans* DNA than treatments ‘Lm only’, ‘Lm + Lb‐3’ and ‘Lm + Lb‐5’ (Fig. 4). It was also that in the treatments ‘Lb + Lm‐1’, ‘Lb + Lm‐3’, ‘Lb + Lm‐5’, as well as the treatment ‘Lm + Lb‐1’, the percentage of *L. maculans* DNA was consistently less than that of ‘Lm&Lb’ (1.9%). There were no significant differences in percentage of *L. maculans* DNA between treatments ‘Lm only’ (100.0%), ‘Lm + Lb‐3’ (97.8%) and ‘Lm + Lb‐5’ (97.9%) (Fig. 4). There were no significant interactions between experiment and treatment (*F*(8,70) = 1.16, *P* = 0.337).

**Figure 4:**
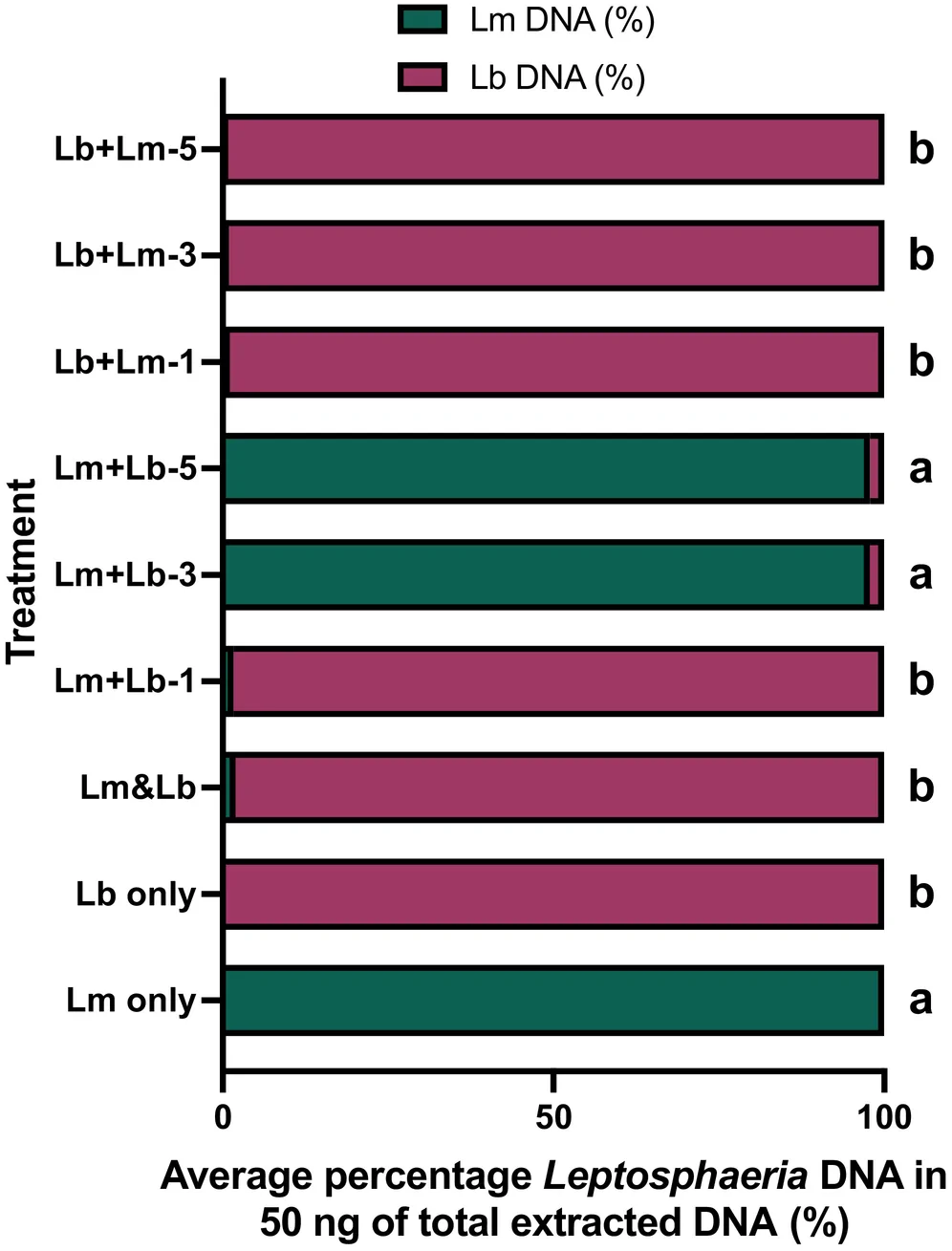
Average percentages of *Leptosphaeria maculans* or *Leptosphaeria biglobosa* DNA (%) in cotyledons from two experiments for different treatments. Cotyledons of oilseed rape (*Brassica napus*) were inoculated with sterilised distilled water (SDW, control), *L. maculans* only (Lm only), *L. biglobosa* only (Lb only), *L. maculans* and *L. biglobosa* co‐inoculated simultaneously (Lm&Lb), initial inoculation with *L. maculans* followed by co‐inoculation with *L. biglobosa* at 1, 3 or 5 days later (Lm + Lb‐1, Lm + Lb‐3, Lm + Lb‐5) or initial inoculation with *L. biglobosa* followed by co‐inoculation with *L. maculans* at 1, 3 or 5 days later (Lb + Lm‐1, Lb + Lm‐3, Lb + Lm‐5). Cotyledons were removed at 16 days post‐inoculation (dpi). Tukey's honestly significant difference (HSD) tests were used to compare the mean percentages of *L. maculans* DNA in total extracted DNA of both *Leptosphaeria* species between different treatments. Treatments that do not share a letter are considered significantly different (*P* < 0.05) (70 df).

### 3.4 Host defence responses to pathogen co‐inoculations

There were significant differences in relative expression of the *PR‐1* gene between different treatments at 2 dpi (*F*(1,3) = 105.20, *P* < 0.0001), 4 dpi (*F*(1,5) = 996.50, *P* < 0.0001) and 6 dpi (*F*(1,5) = 662.80, *P* < 0.0001) (Fig. 5). At 2 dpi, all of the treatments were significantly different from each other, with ‘Lm&Lb’ inducing the greatest up‐regulation of this gene, followed by ‘Lb only’, ‘Lm only’ and ‘SDW’, respectively (Fig. 5(A)). At 4 dpi, there were no significant differences between ‘SDW’, ‘Lm only’ and ‘Lm + Lb‐3’, with all inducing a very small up‐regulation. All of the remaining treatments were significantly different from each other, with ‘Lb only’ inducing the greatest up‐regulation of this gene, followed by ‘Lm&Lb’ and ‘Lb + Lm‐3’, respectively (Fig. 5(B)). At 6 dpi, there were no significant differences between ‘SDW’, ‘Lm only’ and ‘Lm + Lb‐5’, with all inducing a very small up‐regulation. There were no significant differences between ‘Lb only’ and ‘Lm&Lb’, both inducing the greatest up‐regulation of *PR‐1* gene. This was followed by ‘Lb + Lm‐5’, which was significantly different from the rest of the treatments (Fig. 5(C)). Overall, ‘Lb only’ and ‘Lm&Lb’ treatments resulted in the greatest up‐regulation of *PR‐1* at all time points. This up‐regulation was also detected in sequential co‐inoculation treatments where *L. biglobosa* was the first pathogen to be inoculated (Lb + Lm‐3 and Lb + Lm‐5).

**Figure 5:**
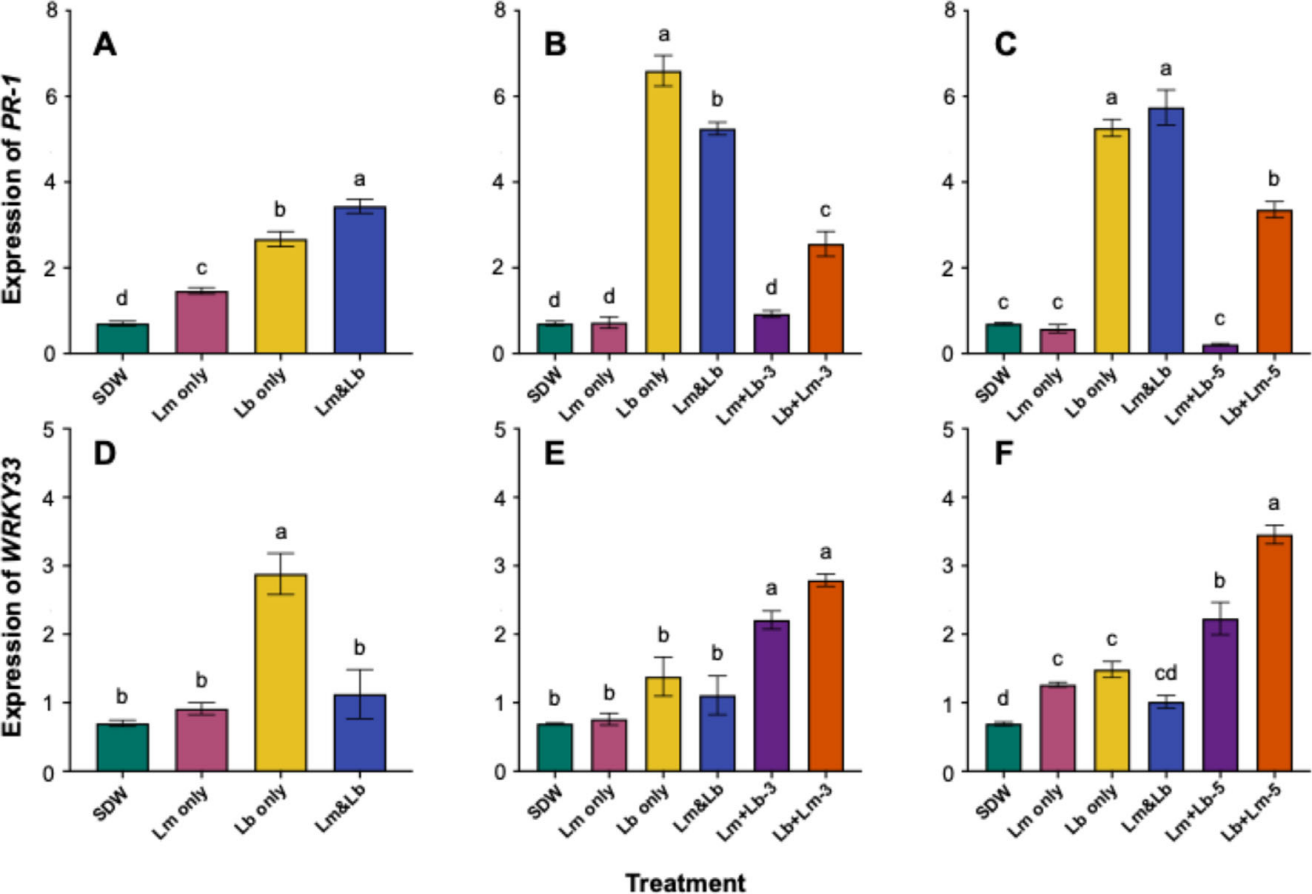
Relative expression of *PR‐1* (A–C) and *WRKY33* (D–F) genes in cotyledons obtained from two experiments for different treatments. Cotyledons of oilseed rape (*Brassica napus*) were inoculated with sterilised distilled water (SDW, control), *Leptosphaeria maculans* only (Lm only), *Leptosphaeria biglobosa* only (Lb only), *L. maculans* and *L. biglobosa* co‐inoculated simultaneously (Lm&Lb), initial inoculation with *L. maculans* followed by co‐inoculation with *L. biglobosa* 3 or 5 days later (Lm + Lb‐3, Lm + Lb‐5) or initial inoculation with *L. biglobosa* followed by co‐inoculation with *L. maculans* 3 or 5 days later (Lb + Lm‐3, Lb + Lm‐5). Sampling for gene expression analysis was done at (A, D) 2 days post‐inoculation (dpi), (B, E) 4 dpi and (C, F) 6 dpi. Tukey's honestly significant difference (HSD) tests were used to compare the mean relative gene expression between different treatments for each gene and sampling point. Treatments that do not share a letter are considered significantly different (*P* < 0.05). Error bars show standard error of the mean (SEM) (*PR‐1* and *WRKY33*: 2 dpi 23 df, 4 dpi 35 df, 6 dpi 35 df each).

There were significant differences in relative expression of the *WRKY33* gene between different treatments at 2 dpi (*F*(1,3) = 347.20, *P* < 0.0001), 4 dpi (*F*(1,5) = 422.60, *P* < 0.0001) and 6 dpi (F(1,5) = 299.90, *P* < 0.0001) (Fig. 5). At 2 dpi, ‘Lb only’ induced the greatest up‐regulation of this gene and this was significantly greater than for all other treatments (Fig. 5(D)). At 4 dpi, ‘Lm + Lb‐3’ and ‘Lb + Lm‐3’ induced the greatest up‐regulation of this gene and these were significantly greater than all other treatments, but not significantly different from each other (Fig. 5(E)). At 6 dpi, ‘Lb + Lm‐5’ induced the greatest up‐regulation of this gene and this was significantly greater than all other treatments. This was followed by ‘Lm + Lb‐5’, which was significantly different to the rest of the treatments. The up‐regulations induced by ‘Lm only’ and ‘Lb only’ were significantly greater than ‘SDW’ but not significantly different to ‘Lm&Lb’ (Fig. 5(F)). Overall, sequential co‐inoculations resulted in the greatest up‐regulation of this gene, especially where *L. biglobosa* was the first pathogen to be inoculated (Lb + Lm‐3 and Lb + Lm‐5). In addition, a similar up‐regulation was observed as a response to ‘Lb only’ inoculation at 2 dpi.

## 4 DISCUSSION

The results of this study provide evidence that the timing of co‐inoculation greatly affects the interspecific interactions between *L. maculans* and *L. biglobosa* in terms of disease severity and relative pathogen growth. For the treatments where *L. biglobosa* was inoculated before *L. maculans*, as well as for simultaneous co‐inoculation of both pathogens, the rates of lesion development were similar to those of *L. biglobosa* only (‘Lb only’). This is consistent with previous work on *L. biglobosa*.^49^, ^50^ Lesion development in these treatments was similar to those in other previous work, where lesions on oilseed rape leaves pretreated with *L. biglobosa* prior to *L. maculans* inoculation developed smaller lesions.^31^, ^36^ Notably, the ‘Lm + Lb‐1’ treatment also showed similar lesions to ‘Lb only’. These observations suggest that *L. biglobosa* may have inhibited the growth of *L. maculans* in those treatments, which is similar to the results of the *in vitro* work.^43^ For the treatments ‘Lm + Lb‐3’ and ‘Lm + Lb‐5’, lesion development was similar to that of Lm only, with little to no lesion development up to 10 dpi, followed by a sudden appearance of relatively large lesions at 14 dpi and continued expansion at 16 dpi. This trend of lesion development is similar to those previously reported for *L. maculans* alone,^49^, ^51^ suggesting that *L. maculans* may have inhibited the growth of *L. biglobosa* in those treatments.

The similar lesion phenotype (small, dark, defined lesion with very few or no pycnidia) and lesion size to ‘Lb only’ for treatments ‘Lb + Lm‐1’, ‘Lb + Lm‐3’, ‘Lb + Lm‐5’ and ‘Lm&Lb’ further suggests that *L. maculans* growth was inhibited. For the ‘Lm + Lb‐1’ treatment, although it had similar lesion phenotype to ‘Lb only’, it had a smaller lesion size, suggesting that while *L. biglobosa* can still inhibit the growth of *L. maculans* locally with a 1‐day timing disadvantage against *L. maculans*, this comes with a cost, resulting in a reduction in lesion severity of *L. biglobosa*. This is most probably due to onset of endophytic colonisation of *L. maculans* at the inoculation site, thereby reducing the availability of nutrients to *L. biglobosa*.^52^ However, the treatments ‘Lm + Lb‐3’ and ‘Lm + Lb‐5’ had similar lesion phenotypes (large, grey, undefined lesion with nature pycnidia) and lesion size to ‘Lm only’, indicating that *L. biglobosa* growth was inhibited in those treatments. The results of lesion phenotypes support the hypothesis that *L. maculans* and *L. biglobosa* can inhibit each other's growth *in planta*, depending on the timing of co‐inoculation, through deployment of interspecific competition strategies, as *in vitro*.^43^ The hypothesis is further supported by results of relative percentages of pathogen DNA in lesions. Little to no *L. maculans* DNA in lesions of treatments ‘Lb + Lm‐1’, ‘Lb + Lm‐3’, ‘Lb + Lm‐5’ and ‘Lm + Lb‐1’ suggests that *L. biglobosa* can inhibit the growth of *L. maculans* if it is inoculated before or up to 1 day after *L. maculans*.

The inhibition of *L. maculans* growth by *L. biglobosa* may be due to the faster metabolic rate of *L. biglobosa* leading to a reduction in nutrients at the inoculation site, reducing and limiting the ability of *L. maculans* to obtain them for growth and development.^43^ An alternative explanation is that the necrotic tissues formed by *L. biglobosa* colonisation are unsuitable for nutrient uptake by *L. maculans* during its early endophytic phase.^52^ Although it is known that *L. maculans* itself is able to cause necrosis on tissues and gather nutrients from them during its late necrotrophic phase, a recent transcriptomic study reported that *L. maculans* may not be able to alter the stages of its lifecycle through adapting its transcriptional programme as a response to local changes caused by *L. biglobosa*.^52^, ^53^ Both of these potential explanations suggest that *L. biglobosa* may be able to deploy a resource‐mediated interspecific competition strategy (creating a local effect through environmental changes and nutrient depletion), in addition to its anticipated host‐mediated interspecific competition strategy (creating a systemic effect through induction of SAR) against *L. maculans*.^1^, ^30^, ^46^ These ideas indicate that *L. biglobosa* may have more forces against *L. maculans* in terms of interspecific competition, which were not considered previously.

At any given sampling point, the greatest up‐regulation in the *PR‐1* gene was induced by ‘Lb only’ or ‘Lm&Lb’ treatments. This is attributed to induction of SAR by the presence of *L. biglobosa*, which is signalled through SA, resulting in the expression of pathogenesis‐related proteins (i.e., *PR‐1*).^36^, ^54^ Moreover, similar results for *PR‐1* up‐regulation as a response to infection by *L. biglobosa* have been reported previously.^31^, ^32^, ^50^ In addition, ‘Lb + Lm‐3’ and ‘Lb + Lm‐5’ treatments resulting in an up‐regulation smaller than ‘Lb only’ or ‘Lm&Lb’ but significantly greater than all other treatments suggests that inhibition of *L. maculans* growth by *L. biglobosa* is most probably facilitated by a combination of both of its resource‐mediated and host‐mediated interspecific competition strategies in local competition *in planta*.

Up‐regulation of *WRKY33* was greatest in sequential co‐inoculation treatments and in ‘Lb only’ treatment at 2 dpi. The latter is not surprising, since *WRKY33* has been reported to be crucial in defence responses against necrotrophic pathogens.^37^, ^38^ Notably, even when *WRKY33* expression was reduced in ‘Lb only’ at 4 dpi and 6 dpi, it was expressed at greater levels in ‘Lb + Lm‐3’ and ‘Lb + Lm‐5’. This suggests that *L. biglobosa* may be increasing its necrotrophic activity, such as secreting cell‐wall degrading enzymes upon detecting *L. maculans*, leading to up‐regulation of *WRKY33*. However, the lack of significant up‐regulation in response to ‘Lm&Lb’ inoculation contradicts this speculation. In addition, at 4 dpi, *WRKY33* expression in response to ‘Lm + Lb‐3’ was similar to that of ‘Lb + Lm‐3’; and at 6 dpi, although the expression of *WRKY33* in response to ‘Lm + Lb‐5’ was smaller than that of ‘Lb + Lm‐5’, it was still greater than that in the other treatments. These suggest that *L. biglobosa* is still recognised as a pathogen by the host even when it is co‐inoculated 3 or 5 days after *L. maculans*.

Interestingly, it was also confirmed here that *L. maculans* was able to inhibit the growth of *L. biglobosa in planta* locally, if it had at least a 3‐day timing advantage over *L. biglobosa*. This is largely similar to the observations from the *in vitro* studies, although with the timeline differing slightly. In this study, there was no inhibition of *L. maculans* in ‘Lm + Lb‐3’ treatment, in contrast to the partial inhibition of *L. maculans* growth *in vitro*.^43^ This might be due to differences in nutrients and several other factors between liquid culture (limited nutrients) and cotyledons of oilseed rape (e.g., plant defence responses). Although the antagonistic effect of *L. maculans* on *L. biglobosa* was attributed to production of sirodesmin PL *in vitro*, it is difficult to ascertain whether it was the same *in planta*. This is due to technical difficulties in investigating production of sirodesmin PL in cotyledons of oilseed rape due to its low level of production.

Another possible explanation for the antagonistic effects of *L. maculans* and *L. biglobosa* is completely dependent on the timing advantage. Although *L. biglobosa* can overcome a 1‐day timing advantage of *L. maculans* (‘Lm + Lb‐1’), it is not able to overcome a three or more days of timing advantage of *L. maculans* (‘Lm + Lb‐3’, ‘Lm + Lb‐5’). These results indicate that the endophytic colonisation of oilseed rape cotyledon tissue by *L. maculans* for at least 3 days may make the local environment unsuitable for *L. biglobosa* to grow and produce lesions. Even though this resembles a resource‐mediated competition strategy (suggested for *L. biglobosa*), it is not deemed to be a direct interspecific competition strategy for *L. maculans* in this study. This is due to the ability of *L. biglobosa* to outcompete *L. maculans* being conferred by its faster metabolic and colonisation rate.^43^, ^55^ However, for *L. maculans* this is completely dependent on inoculation timeline and without a timing advantage of at least 3 days, *L. maculans* is unable to inhibit the growth of *L. biglobosa* locally. Evidently, *L. maculans* was reported to have co‐evolved more strictly with the host compared to *L. biglobosa* ^55^, ^56^ with a narrower host range and has the ability to develop its pseudothecia at colder temperatures, leading to the release of its ascospores earlier than those of *L. biglobosa*.^26^ This has been suggested to allow their co‐existence on one host through occupation of slightly different ecological niches.^9^, ^13^, ^23^ In addition, host defences were reported to be activated as *L. maculans* switched from endotrophy to necrotrophy.^32^, ^52^ Thus, if ascospores of *L. maculans* are released considerably earlier than those of *L. biglobosa* in a growing season, host defences may be activated by the late necrotrophic phase of *L. maculans*, which may reduce the severity of *L. biglobosa* lesions, mimicking induction of SAR. However, ascospores of both *L. maculans* and *L. biglobosa* being released at similar times were observed.^20^ Therefore, out of all the competition strategies discussed, the host‐mediated interspecific competition strategy deployed by *L. biglobosa* is most probable to be observed in natural conditions in the UK. Only one isolate of each pathogen was used in this study. There are seven identified subclades of *L. biglobosa*.^24^, ^57^ There is a need to investigate whether other subclades of *L. biglobosa* have similar interactions with *L. maculans*, especially in regions where both pathogens co‐infect oilseed rape in Europe, Canada and Australia.^23^, ^24^

Similar interactions between other plant pathogens co‐infecting the same host have been observed. For example, pre‐inoculation of barley seedlings with *Puccinia hordei* (leaf rust) reduces the symptom severity of *Erysiphe graminis* (powdery mildew) through reduction of conidia production and biomass. Conversely, pre‐inoculation with *E. graminis* at least 2 days before *Puccinia hordei* reduces the symptom severity of *Puccinia hordei* through reduction of size and amount of rust pustules.^58^ Additionally, wheat leaf spot pathogen *Cochliobolus sativus* can suppress conidial germination of *Pyrenophora tritici‐repentis* on wheat plants when co‐inoculated simultaneously or up to 6 h after *Pyrenophora tritici‐repentis*.^59^

The results of this study have potential practical importance for phoma stem canker management. Over time, through contaminated debris and/or airborne ascospores, *L. maculans* did spread into regions where *L. biglobosa* was previously the dominant pathogen, such as Canada and Poland.^4^, ^6^, ^23^ *Leptosphaeria maculans* became a serious threat to oilseed rape crops in those regions due to favourable environmental factors and its more host‐specialised nature. In the UK, where both pathogens co‐infect oilseed rape, earlier release of *L. biglobosa* ascospores compared to *L. maculans* have been associated with a reduction in the overall stem canker severity score.^20^, ^47^ This study confirmed that interspecific interactions between *L. maculans* and *L. biglobosa* can affect symptom development and subsequent disease severity on their host plants, and that *L. biglobosa* has a greater competitive strength compared to *L. maculans*. Some have suggested the use of *L. biglobosa* as a biocontrol agent against *L. maculans*.^31^, ^32^, ^50^ The results of this study suggest that if the factors (e.g., weather) favour *L. biglobosa*, it might reduce the damage caused by *L. maculans*. Furthermore, timing of ascospore release and disease severity in natural conditions can be affected by various other factors, such as temperature, rainfall, resistance genes and use of fungicides since *L. maculans* and *L. biglobosa* differ in sensitivity to fungicides.^20^, ^47^ Models to predict the timing of ascospore release of *Leptosphaeria* species exist,^11^, ^26^, ^60^ but information available for the earlier‐mentioned factors are limited and there is no distinction made between *L. maculans* and *L. biglobosa*. In order to improve phoma stem canker control, there is a need to develop new models for more specific prediction of *L. maculans* and *L. biglobosa* ascospore release based on biological, genetic and environmental factors and to predict their potential effects on disease epidemics. This could allow a holistic selection of common and novel integrated pest management strategies to provide better control of phoma stem canker on oilseed rape.

## CONFLICT OF INTEREST

There is no conflict of interest to report.

## Data Availability

The data that support the findings of this study are available from the corresponding author upon reasonable request.

## References

[1] Dutt A , Andrivon D and May CL , Multi‐infections, competitive interactions, and pathogen coexistence. Plant Pathol 71:5–22 (2021b). 10.1111/ppa.13469.

[2] Savary S , Willocquet L , Pethybridge SJ , Esker P , McRoberts N and Nelson A , The global burden of pathogens and pests on major food crops. Nat Ecol Evol 3:430–439 (2019). 10.1038/s41559-018-0793-y. 30718852

[3] Zheng X , Koopmann B , Ulber B and von Tiedemann A , A global survey on diseases and pests in oilseed rape—current challenges and innovative strategies of control. Front Agron 2:590908 (2020). 10.3389/fagro.2020.590908.

[4] Zhang X , White RP , Demir E , Jedryczka M , Lange RM , Islam M *et al*., *Leptosphaeria* spp., phoma stem canker and potential spread of *L. maculans* on oilseed rape crops in China. Plant Pathol 63:598–612 (2014). 10.1111/ppa.12146.

[5] CropMonitor , Secure.fera.defra.gov.uk (2022). Retrieved 24 February 2022, from https://secure.fera.defra.gov.uk/cropmonitor/.

[6] Fitt BDL , Hu BC , Li ZQ , Liu SY , Lange RM , Kharbanda PD *et al*., Strategies to prevent spread of *Leptosphaeria maculans* (phoma stem canker) onto oilseed rape crops in China; costs and benefits. Plant Pathol 57:652–664 (2008). 10.1111/j.1365-3059.2008.01841.x.

[7] Stonard JF , Latunde‐Dada AO , Huang YJ , West JS , Evans N and Fitt BDL , Geographic variation in severity of phoma stem canker and *Leptosphaeria maculans/ L. biglobosa* populations on UK winter oilseed rape (*Brassica napus*). Eur J Plant Pathol 126:97–109 (2010). 10.1007/s10658-009-9525-0.

[8] Shoemaker R and Brun H , The teleomorph of the weakly aggressive segregate of *Leptosphaeria maculans* . Can J Bot 79:412–419 (2001). 10.1139/b01-019.

[9] Fitt BDL , Huang YJ , Van Den Bosch F and West JS , Coexistence of related pathogen species on arable crops in space and time. Annu Rev Phytopathol 44:163–182 (2006a). 10.1146/annurev.phyto.44.070505.143417. 16602949

[10] de Gruyter J , Woudenberg J , Aveskamp M , Verkley G , Groenewald J and Crous P , Redisposition of phoma‐like anamorphs in *Pleosporales* . Stud Mycol 75:1–36 (2013). 10.3114/sim0004. PMC3713885 24014897

[11] Huang YJ , Fitt BDL , Jedryczka M , Dakowska S , West JS , Gladders P *et al*., Patterns of ascospore release in relation to phoma stem canker epidemiology in England (*Leptosphaeria maculans*) and Poland (*Leptosphaeria biglobosa*). Eur J Plant Pathol 111:263–277 (2005). 10.1007/s10658-004-4421-0.

[12] Bousset L , Jumel S , Garreta V , Picault H and Soubeyrand S , Transmission of *Leptosphaeria maculans* from a cropping season to the following one. Ann Appl Biol 166:530–543 (2015). 10.1111/aab.12205.

[13] West JS , Balesdent M , Rouxel T , Narcy JP , Huang YJ , Roux J *et al*., Colonization of winter oilseed rape tissues by a/tox+ and B/Tox0 *Leptosphaeria maculans* (phoma stem canker) in France and England. Plant Pathol 51:311–321 (2002a). 10.1046/j.1365-3059.2002.00689.x.

[14] Huang YJ , Toscano‐Underwood C , Fitt BDL , Hu XJ and Hall AM , Effects of temperature on ascospore germination and penetration of oilseed rape (*Brassica napus*) leaves by A‐ or B‐group *Leptosphaeria maculans* (phoma stem canker). Plant Pathol 52:245–255 (2003). 10.1046/j.1365-3059.2003.00813.x.

[15] Huang YJ , Li Z , Evans N , Rouxel T , Fitt BDL and Balesdent M , Fitness cost associated with loss of the *AvrLm4* avirulence function in *Leptosphaeria maculans* (phoma stem canker of oilseed rape). Eur J Plant Pathol 114:77–89 (2006). 10.1007/s10658-005-2643-4.

[16] Huang YJ , Pirie EJ , Evans N , Delourme R , King GJ and Fitt BDL , Quantitative resistance to symptomless growth of *Leptosphaeria maculans* (phoma stem canker) in *Brassica napus* (oilseed rape). Plant Pathol 58:314–323 (2009). 10.1111/j.1365-3059.2008.01957.x.

[17] West JS , Fitt BDL , Leech PK , Biddulph JE , Huang YJ and Balesdent M , Effects of timing of *Leptosphaeria maculans* ascospore release and fungicide regime on phoma leaf spot and phoma stem canker development on winter oilseed rape (*Brassica napus*) in southern England. Plant Pathol 51:454–463 (2002b). 10.1046/j.1365-3059.2002.00726.x.

[18] Kerdraon L , Barret M , Balesdent M‐H , Suffert F , Laval Kerdraon L , Barret M *et al*., Impact of a resistance gene against a fungal pathogen on the plant host residue microbiome: the case of the *Leptosphaeria maculans–Brassica napus* pathosystem. Mol Plant Pathol 21:1545–1558 (2020). 10.1111/mpp.12994. PMC7694673 32975002

[19] Jacques N , Balesdent M , Rouxel T and Laval V , New specific quantitative real‐time PCR assays shed light on the epidemiology of two species of the *Leptosphaeria maculans–Leptosphaeria biglobosa* species complex. Plant Pathol 70:643–654 (2021). 10.1111/ppa.13323.

[20] Huang YJ , Sidique SNM , Dewage CSK , Gajula LH , Mitrousia GK , Qi A *et al*., Effective control of *Leptosphaeria maculans* increases importance of *L. biglobosa* as a cause of phoma stem canker epidemics on oilseed rape. Pest Manag Sci 80:2405–2415 (2024). 10.1002/ps.7248. 36285624

[21] Liu Z , Latunde‐Dada AO , Hall AM and Fitt BDL , Phoma stem canker disease on oilseed rape (*Brassica napus*) in China is caused by *Leptosphaeria biglobosa* ‘brassicae.’. Eur J Plant Pathol 140:841–857 (2014). 10.1007/s10658-014-0513-7.

[22] Cai X , Huang YJ , Jiang D , Fitt BD , Li G and Yang L , Evaluation of oilseed rape seed yield losses caused by *Leptosphaeria biglobosa* in central China. Eur J Plant Pathol 150:179–190 (2017). 10.1007/s10658-017-1266-x.

[23] Fitt BDL , Brun H , Barbetti MJ and Rimmer SR , World‐wide importance of phoma stem canker (*Leptosphaeria maculans* and *L. biglobosa*) on oilseed rape (*Brassica napus*). Eur J Plant Pathol 114:3–15 (2006). 10.1007/s10658-005-2233-5.

[24] Dilmaghani A , Balesdent MH , Didier JP , Wu C , Davey J , Barbetti MJ *et al*., The *Leptosphaeria maculans* – *Leptosphaeria biglobosa* species complex in the American continent. Plant Pathol 58:1044–1058 (2009). 10.1111/j.1365-3059.2009.02149.x.

[25] Abdullah AS , Moffat CS , Lopez‐Ruiz FJ , Gibberd MR , Hamblin J and Zerihun A , Host–multi‐pathogen warfare: pathogen interactions in co‐infected plants. Front Plant Sci 8:1806 (2017). 10.3389/fpls.2017.01806. PMC5660990 29118773

[26] Toscano‐Underwood C , Huang YJ , Fitt BDL and Hall AM , Effects of temperature on maturation of pseudothecia of *Leptosphaeria maculans* and *L. biglobosa* on oilseed rape stem debris. Plant Pathol 52:726–736 (2003). 10.1111/j.1365-3059.2003.00930.x.

[27] Tan S , Gu Y , Yang C , Dong Y , Mei X , Shen Q *et al*., *Bacillus amyloliquefaciens* T‐5 may prevent *Ralstonia solanacearum* infection through competitive exclusion. Biol Fertil Soils 52:341–351 (2016). 10.1007/s00374-015-1079-z.

[28] Dutt A , Anthony R , Andrivon D , Jumel S , Roy GL , Baranger A *et al*., Competition and facilitation among fungal plant parasites affect their life‐history traits. Oikos 130:652–667 (2021a). 10.1111/oik.07747.

[29] Aimé S , Alabouvette C , Steinberg C and Olivain C , The endophytic strain *fusarium oxysporum* FO47: a good candidate for priming the defense responses in tomato roots. Mol Plant Microbe Interact 26:918–926 (2013). 10.1094/mpmi-12-12-0290-r. 23617416

[30] Mahuku G , Hall R and Goodwin P , Co‐infection and induction of systemic acquired resistance by weakly and highly virulent isolates of *Leptosphaeria maculans* in oilseed rape. Physiol Mol Plant Pathol 49:61–72 (1996). 10.1006/pmpp.1996.0039.

[31] Liu S , Liu R , Latunde‐Dada AO , Cools HJ , Foster SJ , Huang YJ *et al*., Comparison of *Leptosphaeria biglobosa*‐induced and chemically induced systemic resistance to *L. maculans* in *Brassica napus* . Chin Sci Bull 52:1053–1062 (2007). 10.1007/s11434-007-0181-5.

[32] Padmathilake KRE and Fernando WGD , Less virulent *Leptosphaeria biglobosa* immunizes the canola plant to resist highly virulent *L. maculans*, the blackleg pathogen. Plants 11:996 (2022). 10.3390/plants11070996. PMC9002471 35406977

[33] Li N , Han X , Feng D , Yuan D and Huang L , Signaling crosstalk between salicylic acid and ethylene/jasmonate in plant defense: do we understand what they are whispering? Int J Mol Sci 20:671 (2019). 10.3390/ijms20030671. PMC6387439 30720746

[34] Shafqat A , Abbas S , Ambreen M , Bhatti AS , Kausar H and Gull T , Exploring the vital role of phytohormones and plant growth regulators in orchestrating plant immunity. Physiol Mol Plant Pathol 133:102359 (2024). 10.1016/j.pmpp.2024.102359.

[35] Dong X , SA, JA, ethylene, and disease resistance in plants. Curr Opin Plant Biol 1:316–323 (1998). 10.1016/1369-5266(88)80053-0. 10066607

[36] Durrant W and Dong X , Systemic acquired resistance. Annu Rev Phytopathol 42:185–209 (2004). 10.1146/annurev.phyto.42.040803.140421. 15283665

[37] Zheng Z , Qamar SA , Chen Z and Mengiste T , *Arabidopsis* WRKY33 transcription factor is required for resistance to necrotrophic fungal pathogens. Plant J 48:592–605 (2006). 10.1111/j.1365-313x.2006.02901.x. 17059405

[38] Birkenbihl RP , Diezel C and Somssich IE , *Arabidopsis* WRKY33 is a key transcriptional regulator of hormonal and metabolic responses toward *Botrytis cinerea* infection. Plant Pathology 159:266–285 (2012). 10.1104/pp.111.192641. PMC3375964 22392279

[39] Elliott CE , Gardiner DM , Thomas G , Cozijnsen A , Van De Wouw A and Howlett BJ , Production of the toxin sirodesmin PL by *Leptosphaeria maculans* during infection of *Brassica napus* . Mol Plant Pathol 8:791–802 (2007). 10.1111/j.1364-3703.2007.00433.x. 20507539

[40] Rouxel T , Chupeau Y , Fritz R , Kollmann A and Bousquet J , Biological effects of sirodesmin PL, a phytotoxin produced by *Leptosphaeria maculans* . Plant Sci 57:45–53 (1988). 10.1016/0168-9452(88)90140-9.

[41] Gardiner DM , Cozijnsen AJ , Wilson LM , Pedras MSC and Howlett BJ , The sirodesmin biosynthetic gene cluster of the plant pathogenic fungus *Leptosphaeria maculans* . Mol Microbiol 53:1307–1318 (2004). 10.1111/j.1365-2958.2004.04215.x. 15387811

[42] Fortune JA , Bingol E , Qi A , Baker D , Ritchie F , Dewage CSK *et al*., *Leptosphaeria biglobosa* inhibits the production of sirodesmin PL by *L. maculans* . Pest Manag Sci 80:2416–2425 (2024). 10.1002/ps.7275. 36327145

[43] Bingol E , Qi A , Karandeni‐Dewage C , Ritchie F , Fitt BDL and Huang YJ , Co‐inoculation timing affects the interspecific interactions between phoma stem canker pathogens *Leptosphaeria maculans* and *Leptosphaeria biglobosa* . Pest Manag Sci 80:2443–2452 (2024). 10.1002/ps.7799. 37759352

[44] Huang YJ , Mitrousia GK , Sidique SNM , Qi A and Fitt BDL , Combining *R* gene and quantitative resistance increases effectiveness of cultivar resistance against *Leptosphaeria maculans* in *Brassica napus* in different environments. PLoS One 13:e0197752 (2018). 10.1371/journal.pone.0197752. PMC5965857 29791484

[45] Inglis PW , De Castro R Pappas M , Resende LV and Grattapaglia D , Fast and inexpensive protocols for consistent extraction of high quality DNA and RNA from challenging plant and fungal samples for high‐throughput SNP genotyping and sequencing applications. PLoS One 13:e0206085 (2018). 10.1371/journal.pone.0206085. PMC6193717 30335843

[46] Liu SY , Liu Z , Fitt BDL , Evans N , Foster SJ , Huang YJ *et al*., Resistance to *Leptosphaeria maculans* (phoma stem canker) in *Brassica napus* (oilseed rape) induced by *L. biglobosa* and chemical defence activators in field and controlled environments. Plant Pathol 55:401–412 (2006). 10.1111/j.1365-3059.2006.01354.x.

[47] Huang YJ , Hood JR , Eckert MR , Stonard JF , Cools HJ , King GJ *et al*., Effects of fungicide on growth of *Leptosphaeria maculan*s and *L. biglobosa* in relation to development of phoma stem canker on oilseed rape (*Brassica napus*). Plant Pathol 60:607–620 (2011). 10.1111/j.1365-3059.2010.02416.x.

[48] Livak KJ and Schmittgen TD , Analysis of relative gene expression data using real‐time quantitative PCR and the 2−ΔΔCT method. Methods 25:402–408 (2001). 10.1006/meth.2001.1262. 11846609

[49] Lowe RGT , Cassin A , Grandaubert J , Clark BL , Van De Wouw AP , Rouxel T *et al*., Genomes and transcriptomes of partners in plant‐fungal‐ interactions between canola (*Brassica napus*) and two *Leptosphaeria* species. PLoS One 9:e103098 (2014). 10.1371/journal.pone.0103098. PMC4113356 25068644

[50] Shah UA , Kotta‐Loizou I , Fitt BDL and Coutts RHA , Mycovirus‐induced hypervirulence of *Leptosphaeria biglobosa* enhances systemic acquired resistance to *Leptosphaeria maculans* in *Brassica napus* . Mol Plant Microbe Interact 33:98–107 (2020). 10.1094/mpmi-09-19-0254-r. 31652089

[51] Haddadi P , Larkan NJ and Borhan MH , Dissecting *R* gene and host genetic background effect on the *Brassica napus* defense response to *Leptosphaeria maculans* . Sci Rep 9:6947 (2019). 10.1038/s41598-019-43419-9. PMC6502879 31061421

[52] Gay EJ , Jacques N , Lapalu N , Cruaud C , Laval V , Balesdent M *et al*., Location and timing govern tripartite interactions of fungal phytopathogens and host in the stem canker species complex. BMC Biol 21:247 (2023). 10.1186/s12915-023-01726-8. PMC10631019 37936151

[53] Gay EJ , Soyer JL , Lapalu N , Linglin J , Fudal I , Da Silva C *et al*., Large‐scale transcriptomics to dissect 2 years of the life of a fungal phytopathogen interacting with its host plant. BMC Biol 19:55 (2021). 10.1186/s12915-021-00989-3. PMC7986464 33757516

[54] Ali S , Mir ZA , Bhat JA , Tyagi A , Chandrashekar N , Yadav P *et al*., Isolation and characterization of systemic acquired resistance marker gene *PR1* and its promoter from *Brassica juncea* . 3 Biotech 8:10 (2017). 10.1007/s13205-017-1027-8. PMC5723574 29259885

[55] Frąc M , Kaczmarek J and Jędryczka M , Metabolic capacity differentiates *Plenodomus lingam* from *P. Biglobosus* subclade ‘brassicae’, the causal agents of phoma leaf spotting and stem canker of oilseed rape (*Brassica napus*) in agricultural ecosystems. Pathogens 11:50 (2022). 10.3390/pathogens11010050. PMC8778923 35055998

[56] Grandaubert J , Lowe RG , Soyer JL , Schoch CL , Van De Wouw AP , Fudal I *et al*., Transposable element‐assisted evolution and adaptation to host plant within the *Leptosphaeria maculans‐Leptosphaeria biglobosa* species complex of fungal pathogens. BMC Genomics 15:891 (2014). 10.1186/1471-2164-15-891. PMC4210507 25306241

[57] Zou Z , Zhang X , Parks P , Du Toit LJ , Van De Wouw AP and Fernando WGD , A new subclade of *Leptosphaeria biglobosa* identified from *Brassica rapa* . Int J Mol Sci 20:1668 (2019). 10.3390/ijms20071668. PMC6479289 30987176

[58] Round PA and Wheeler BEJ , Interactions of *Puccinia hordei* and *Erysiphe graminis* on seedling barley. Ann Appl Biol 89:21–35 (1978). 10.1111/j.1744-7348.1978.tb02563.x.

[59] da Luz WC , Interactions between *Cochliobolus sativus* and *Pyrenophora tritici‐ repenti*s on wheat leaves. Phytopathology 77:1355 (1987). 10.1094/phyto-77-1355.

[60] Salam MU , Fitt BDL , Aubertot J , Diggle AJ , Huang YJ , Barbetti MJ *et al*., Two weather‐based models for predicting the onset of seasonal release of ascospores of Leptosphaeria maculans or L. biglobosa. Plant Pathol 56:412–423 (2007). 10.1111/j.1365-3059.2006.01551.x.

